# Charitable Giving in Times of Covid-19: Do Crises Forward the Better or the Worse in Individuals?

**DOI:** 10.1007/s11266-023-00558-y

**Published:** 2023-02-28

**Authors:** Julia Litofcenko, Michael Meyer, Michaela Neumayr, Astrid Pennerstorfer

**Affiliations:** 1 Institute for Nonprofit Management, WU Vienna, University of Economics and Business, Welthandelsplatz 1, 1020 Vienna, Austria; 2 Institute for Social Policy, WU Vienna, University of Economics and Business, Welthandelsplatz 1, 1020 Vienna, Austria

**Keywords:** Charitable giving, Covid-19, Terror management theory, Austria, Germany

## Abstract

Why did some individuals react to the Covid-19 crisis in a prosocial manner, whereas others withdrew from society? To shed light onto this question, we investigate changing patterns of charitable giving during the pandemic. The study analyzes survey data of 2000 individuals, representative of the populations of Germany and Austria. Logistic regressions reveal that personal affectedness by Covid-19 seems to play a crucial role: those who were personally affected either mentally, financially, or health-wise during the first 12 months of Covid-19 were most likely to have changed their giving behavior. The observed patterns fit psychological explanations of how human beings process existential threats. Our findings indicate that a profound societal crisis in itself mainly leads to changes in charitable giving if individuals are severely affected on a personal level. Thereby, we contribute to a better understanding of the mechanisms underlying individuals’ charitable giving behavior in times of crisis.

**Supplementary Information:**

The online version contains supplementary material available at 10.1007/s11266-023-00558-y.

## Introduction

The outbreak of a novel coronavirus (Covid-19) in the early spring of 2020 led to a crisis worldwide. Millions of people lost their jobs, more than 600 million got infected, and more than 6.6 million people died with Covid-19 by the end of 2022 (World Health Organization, 2020). In many countries, governments were overwhelmed with multiple challenges, and nonprofit organizations (NPOs) and grassroots initiatives stepped in trying their best to provide flexible solutions to the needs of the population—and to prevent those who needed help most from being left unserved. Since people with lower socioeconomic status have been particularly affected by the negative consequences of the pandemic (e.g., Auriemma & Iannaccone, 2020), NPOs played an important role in mitigating this inequality. Their quick and non-bureaucratic services were in high demand (Meyer et al., 2021), but they relied on monetary donations to accomplish this challenge.

Individuals’ commitment to charitable giving was anything but "normal" in these exceptional times, as the pandemic also impacted people's civic engagement. Two opposing effects seem to have operated simultaneously. On the one hand, donors withdrew from previous engagements, and nonprofits reported stalled donations, limiting their scope for action (CAF, 2021). Presumably, the pandemic aggravated financial inequalities, and individuals from low-income groups were more affected by unemployment or illness, thus less able to donate. In addition, the pandemic triggered financial insecurity and uncertainty more generally because many people or people close to them were affected by unemployment or illness. Images in the media permanently showing medical staff in hospitals struggling with a lack of protective equipment, running out of oxygen, and reaching their capacity limits exacerbated public tension even further, causing fear (of death), anxiety, and irritation in many people (Sin et al., 2021). To cope with this uncertainty, people made unreasonably large panic purchases of items such as toilet paper, which can be “*interpreted as a selfish way of coping with insecurity, uncertainty and loss of control*” (Jin & Ryu, 2021, p. 416). The fear of death and the resulting existential insecurity can bring out a self-preserving way of coping in people—which includes cancelling charitable donations.

On the other hand, the pandemic has also brought out the better in people. During the first months of the crisis, the news frequently reported cases of spontaneous prosocial behavior. Donation portals by communities and other forms of assistance (e.g., telephone helplines against social isolation) arose, and solidarity and informal helping behavior increased tremendously. In these cases, situations that remind individuals of their mortality lead to an increase in prosocial, benevolent behaviors (Burke et al., 2010; Henrich, 2020)—including charitable donations.

Given these opposing effects, why did some people get involved and helped others while their own lives were in danger amid the Covid-19 crisis, and why did others quit their civic engagement? In aiming to understand the psychological mechanisms that affect changes in charitable giving, we draw onto terror management theory (TMT), a stream of research investigating how individuals react to existential threats. Based on this literature, we develop hypotheses that link being personally affected by the Covid-19 crisis—in mental, financial, and health-wise terms—to changes in charitable giving.

We analyze survey data from 2000 individuals, representative of the populations in Germany and Austria, collected between September and October 2021. As charitable giving we understand all voluntary contributions of money to organizations that provide benefits to others (Bernholz et al., 2016). We do not include donations of time, since the patterns regarding volunteering were highly dependent on curfews and other legal restrictions, thus reflecting personal decisions to a much lower degree.

Our study’s contribution is twofold: First, we transfer the concepts of TMT from experimental to survey data. Previous studies have, to our knowledge, investigated the effect of existential threats on prosocial behavior in experimental settings only. Second, and more important, by connecting insights from philanthropy research to psychological theories on human responses to crises, we contribute to a better understanding of the mechanisms underlying individuals’ giving behavior in times of crisis. This seems to be of great importance, as crisis seems so be the normal state, with the financial crisis of 2008, the refugee crisis in Europe after 2015, the yet ongoing crisis of democracy and the looming climate collapse haunting our societies.

## Prior Research and Theory

Donating money is a widespread form of civic engagement. According to the 160-country World Gallup Polls, one in three adults worldwide gives money to charitable purposes (CAF, 2021), with a considerable variation in the participation rates across countries. Longitudinal studies show that the rates within countries do not fluctuate significantly over time. Notable changes in giving behavior occur only in response to disasters and humanitarian crises. For example, in the US a sharp increase in the volumes of donations was visible directly after the 2010 Haitian earthquake or the 2004 Southeast Asia earthquake resulting in a tsunami (Kapucu, 2016). Such increase in times of disaster is often explained in terms of cooperative economic behavior. But is a pandemic that spreads across the world comparable?

Individual giving behavior is a well-studied terrain (e.g., Bekkers & Wiepking, 2011). We know that the likelihood and the amount of money donated depend on factors on the individual and contextual levels. Factors on the individual level include personal dispositions such as traits, attitudes, norms and values (e.g., altruism, empathic concern, and religiosity), and resources like educational attainment, income, or the extent and quality of social networks (e.g., Bekkers & Wiepking, 2011). On the contextual level, which has been less systematically studied due to a lack of comparative data, factors include legal regulations and fiscal incentives (‘formal institutions’), cultural or religious norms (‘informal institutions’, see Wiepking et al., 2021), but also situational factors (Von Schnurbein et al., 2021). Among the situational drivers that are said to increase donation behavior, the "being asked" is the most relevant, because the vast majority of donations are made because people have been specifically asked to donate. Further drivers of giving are an individual’s reputation, i.e., giving money in a situation where other people are watching, the awareness of need, the efficacy of giving, its costs, and psychological benefits donors receive when giving money (Bekkers & Wiepking, 2011).

Hitherto, research on charitable giving related to Covid-19 has mostly discussed perceived needs and effectiveness as factors that alter giving behavior. Perceived needs refer to the awareness of need, which is described as a prerequisite of charitable giving (Bekkers & Wiepking, 2011). In case of Covid-19, higher awareness of need is fostered by the ubiquity of the pandemic in mass media, but also because many people personally know victims of Covid-19. Media coverage also makes the need more salient and creates identifiable victims by showing images of real people suffering, thereby reducing the social distance between donors and potential recipients (Smith, 2022). Media coverage is considered a key factor in explaining donation responses to disasters (Eisensee & Strömberg, 2007), and changes in the awareness of need can increase the donor pool as well as the amounts donated, or evoke a change in causes people donate money for. Overall, the awareness of need is assumed to increase with education as well as with the proximity to the targeted issues (Lim & DeSteno, 2016; Piff et al., 2010).

Based on data from UK, Zagefka (2021) finds that the way how a person appreciates the strategies to fight the pandemic—either sharing a global common fate in overcoming the crisis or rather ‘closing ranks’ at the national level—influences to what groups she donates to. In a similar vein, Abel and Brown (2020) discuss how public and private positive or negative role models in fighting the pandemic increase trust and responsibility, which in turn lead to increased donations to emergency response. Zhou et al. (2021) discuss how different individual cognitive styles and the perception of efficacy impact giving to Covid-19-related causes.

Theoretical frames that substantiate such findings and explain how crises modify philanthropic behavior are rare. One of the theories that may fill this gap and explain changes in giving behavior in the context of the Covid-19 pandemic is terror management theory (TMT) (see Albouy, 2014; Pyszczynski et al., 2021). TMT delineates a stream of psychological research and explains how individuals react to threatening situations, which the Covid-19 pandemic clearly was. It massively triggered existential threats for many people (Burke et al., 2010; Courtney et al., 2020). Originating in *Denial of Death* by Ernest Becker (1973), TMT hypothesizes that we constantly need to manage our awareness of death due to the nature of our human consciousness. We overcome this existential insecurity by socially constructing beliefs that give meaning, self-esteem, and predictability to life, thereby setting us apart from our animal nature (Greenberg et al., 1986; Hayes et al., 2010).

TMT differentiates two levels of coping strategies with which individuals react to reminders of existential insecurity: *Proximal defenses* refer to immediate threat-focused attempts to push death out of our consciousness. In this mode, individuals try to escape or solve the threatening situation, to regain some safer ground. If this is successful, one enters the second level of coping, in which the so-called *distal defenses* operate. Distal defense mechanisms induce a striving for symbolic immortality: to be part of and contribute to something greater than oneself that lasts forever (Greenberg et al., 1990; Pyszczynski et al., 1999; Rosenblatt et al., 1989). Symbolic immortality is approached by validating the cultural worldview, enhancing one’s self-esteem, and strengthening close relationships. Through these behaviors, individuals try to protect themselves against existential fragility and retain psychological equanimity (Zaleskiewicz et al., 2015).

Proximal defenses, aimed at pushing the immediate threat out of our consciousness, usually induce self-defensive behavior. The urge to defend oneself in the face of existential threats leads to increased support for the punishment of social transgressions (Florian & Mikulincer, 1997; Rosenblatt et al., 1989), and a polarization between in- and out-groups, with adverse reactions toward the out-group (Greenberg et al., 1986). Roberts and Maxfield (2019) document decreases in charitable giving among younger age groups in the wake of existential threats, likely driven by worries about financial security in the future.

When the immediate threat is overcome, which means the threat is not consciously present any more, individuals enter the state of distal defense mechanisms. Distal defenses drive prosocial, benevolent behaviors. Individuals want to validate their cultural worldview, and prosocial acts constitute the foundations of most, if not all, cultural worldviews (Burke et al., 2010; Henrich, 2020). Thus, prosocial behavior—such as charitable giving—as a reaction to existential threats appears when the immediate threat for one’s life is overcome.

Empirical studies support a moderate yet significant effect of existential threats on prosocial behavior in general (for an overview, see Burke et al., 2010). In particular, Bruine de Bruin and Ulqinaku (2021), Jonas et al. (2002), Jonas et al. (2013), and Jin and Ryu (2021) show that people increase their philanthropic engagement when facing existential threats. Individuals become more generous after they have reflected on their mortality (Jonas et al., 2002). Burke et al. (2010), Roberts and Maxfield (2019), and Cutler et al. (2021) suggest age as a moderating variable between existential threats and the willingness to donate. Younger adults react with self-defensiveness to existential threats, whereas older adults react with an increase in prosocial behavior. Older adults might also donate preferably to in-group causes, which is explained by a desire to care for the survival of the in-group (Cutler et al., 2021; Roberts & Maxfield, 2019).

Studies examining effects of existential threats in the context of Covid-19 show how this threat increased anxiety of individuals—both about one’s own death and general anxiety—which, in turn, had an impact on prosocial behavior. Jin and Ryu (2021) show that people donate more when facing existential threats. Likewise, Grimalda et al. (2021) found that personal exposure to Covid-19 increased donations and argue that situations of existential threat foster individuals’ prosocial attitudes.

Altogether, TMT’s explanations that personal exposure to a situation of existential threat and the possible options to cope with it provide an appropriate framework for our research questions. Prior research using TMT, however, is exclusively experimental. We still miss survey-based research that investigates these effects under real-life conditions.

**Hypotheses** Based on prior research and on the assumptions of TMT, we hypothesize that individuals’ responses to the Covid-19 crisis regarding charitable giving depend on whether and how they were affected by the crisis. We assume that individuals who were most negatively affected are the ones most likely to have changed their giving behavior. Nevertheless, people can be affected by the pandemic in different ways. First, we focus on mental affliction, which is connected to death anxiety. We assume that people who experienced the highest levels of death anxiety due to the pandemic changed their donation behavior with the highest likelihood.

**H1**: Individuals that were most negatively affected by the pandemic mentally were most likely to change (i.e., increase or decrease) their giving behavior compared to those that were less negatively affected mentally.

Whether individuals increased or decreased their donations depends on whether they were able to push the immediate threat posed by the pandemic out of their consciousness. For instance, individuals who experienced high levels of threat throughout the first year of the pandemic and could not suppress anxiety because it was constantly re-triggered by the mass media are more likely to decrease their donations. According to TMT, such a mental condition induces self-defensive behavior, thus leading to reductions in donations (Courtney et al., 2020; Jin & Ryu, 2021; Pyszczynski et al., 1999; Roberts & Maxfield, 2019). On the other hand, individuals who cope with their immediate threat and overcome their anxiety might be more likely to increase their charitable giving.

Many people were not (only) concerned mentally, but also financially. For example, they were put on short-time work or became unemployed—resulting in a reduction in income. Loss of income can lead to existential threat and, according to TMT, to a reduction in charitable giving. On the other hand, individuals could also have increased charitable giving, because those most negatively affected are those who most likely perceive the need to donate in the current situation (Piff et al., 2010). Moreover, those individuals have a higher need for the warm glow associated with charitable behavior (Bekkers & Wiepking, 2011) and a greater need to validate their social status (Burke et al., 2010; Greenberg et al., 1986; Jonas et al., 2002). We thus hypothesize:

**H2**: Individuals that were most negatively affected by the pandemic in their financial well-being were most likely to change (i.e., increase or decrease) their giving behavior compared to those that were less negatively affected in their financial well-being.

Analogously to the effects on mental affection, the direction of the change depends on whether proximal or distal defenses prevail.

Of course the Covid-19 pandemic also affected many people's physical health. As no vaccine was available in the first phase of the pandemic, many people got hospitalized, in many cases with long-lasting health restrictions. In-line with our first two hypotheses, H3 reads:

**H3**: Individuals that were most negatively affected by the pandemic health-wise were most likely to change (i.e., increase or decrease) their giving behavior compared to those that were less negatively affected health-wise.

Here, too, the change may be possible in both directions. Either people affected by health issues donate more because they can empathize better with the situation of others. Or the affectedness went hand in hand with existential fears about the future, and they donated less for that very reason.

## Data and Method

We collected data on charitable giving in Austria and Germany, countries with a social-democratic and corporatist tradition. While around half of the population in Germany reports giving money to nonprofit organizations in a 12-month period, it is two-thirds of the population in Austria (Neumayr & Schober, 2012; Wilke, 2020). Although large parts of the population donate, individuals mostly donate small amounts. The average donation per capita per year is 100 Euro in Austria and 150 Euro in Germany, while it is 270 Euro in the UK and 1240 Euro in the US (FVA, 2022). Compared to countries with a liberal welfare tradition, donations in both Austria and Germany are perceived more as a supplement to state funding, as the majority of the population assumes that the state is responsible for those in need.

In the pandemic, however, the government was overwhelmed and relied heavily on the help of nonprofits. Governments have long since cooperated closely with some of them, which provide many social and health-related services. Citizens were well aware of the importance of nonprofits’ services to cope with the consequences of the pandemic.

Many of them donated through the newly established online donation appeals that some nonprofits quickly developed, as face-to-face fundraising, which had previously been very important in Austria and Germany, was no longer possible due to curfews (Global Philanthropy Environment Index Full Report, 2018; Salamon & Anheier, 1998).

Our study uses a representative sample of 1000 Austrian and 1000 German residents (*n* = 2000) who participated in a comprehensive online survey on philanthropic behavior prior to and since the Covid-19 outbreak. We collected the data together with the Austrian Gallup Institute in September and October 2021. The Austrian Gallup Institute maintains a pool of potential participants who can be contacted for online surveys, which was used to recruit participants representative of the German and Austrian population, respectively, regarding age, gender, highest level of education, occupation, region (federal state), and size of community. We asked questions about individual charitable giving and how the Covid-19 pandemic affected respondents’ physical health, financial situation, mental well-being, life satisfaction, and anxiety levels. Additionally, we asked some general questions regarding the respondents' attitudes (e.g., generalized social trust, and altruism). All questions that were used for the models presented in this paper are available in English translation as online supplementary material.

To test the hypotheses, we estimate binary logistic regressions and analyze which factors cause individuals to change their giving behavior. We compare three groups of individuals: those who did not change, those who increased charitable giving and those who decreased charitable giving. Although we have three groups, we use multiple binary logistic regression models, as the Brant test was rejected (Brant, 1990). This test examines whether the assumptions for parallelism hold, a condition for performing ordered logistic regression. This indicates that the factors explaining differences over the groups are not symmetric, i.e., increases in charitable giving are caused by other factors than decreases. Table 1 displays the results of four models. Model 1 and Model 3 compare individuals with increased charitable giving to persons who have not changed their giving behavior, and Models 2 and 4 compare individuals with decreased charitable giving to individuals who have not changed their giving. In a second step, and in order to scrutinize differences between the increasing and decreasing group, we directly compared these two groups using a reduced sample that consisted only of individuals who displayed changed charitable giving (see Table 2).

To avoid multicollinearity, we estimated separate models for two of the three variables of interest, i.e., financial and health-wise affliction. To ensure linearity between the logit and the metric independent variables, we performed Box-Tidwell tests. None yielded significant coefficients for the logarithmic transformation of any independent metric factor, indicating that it is plausible to assume linearity. To check for model misspecification, we inspected the chi-square test for overall model significance, McFadden’s, Cox and Snell’s as well as Nagelkerke’s Pseudo-*R*^2^, and considered the Link-test for model misspecification (Hair et al., 2019). Among these test-statistics, all indicated a good fit of the model to the data. However, the Link-test showed incidence for model misspecification in Models 1 and 3. Salas-Eljatib et al. (2018) show that this is common when working with unbalanced data, as is the case in these models, and thus the results of the Link-test can only be interpreted with caution.

Two different dependent variables are used to describe individual changes in donation behavior due to the Covid-19 pandemic, “increase in donations (yes/no)” and “decrease in donations (yes/no)”. In the survey, respondents were asked about their charitable giving in the twelve months before the outbreak of the Covid-19 pandemic, i.e., March 2019 to February 2020, and about their charitable giving in the twelve months after the outbreak, which is March 2020 to February 2021. Changes in the incidence and amount between these two periods are taken together. Consequently, “increase in donations” implies that the respondent either started to donate during Covid-19 when she did not donate before, or that she increased the amount donated, and vice versa for “decrease in donations”. More than three-quarters of the respondents in Austria and Germany (77.2%) did not change their charitable giving behavior, while 12% increased and 10.7% decreased donations. Table 3 in "Appendix" displays disaggregated results for changes in incidence and amount. As can be seen in this table, the relatively low number of observations in the group displaying a change in behavior precluded separate analyses of changes in terms of incidence and amount.

In accordance with our hypotheses, analyses include three independent variables that capture how a person was affected by the Covid-19 pandemic. For this, respondents were asked to rate how they were personally affected financially and health-wise on an eleven-point Likert scale ranging from 0 (very positively) to 10 (very negatively). Furthermore, we make use of an item battery that captures how an individual was affected mentally. In total, ten items are combined,^1^^,^^2^ for which respondents were asked to assess different statements on a five-point Likert scale from 1 (never) to 5 (permanently). This combined index was constructed by dividing the sum of all responses by the number of questions. Of this combined index, the first five items represent the death anxiety scale (DAS), which is a set of attitudes that capture vague feelings of fear and unease generated by perceptions of a real or imagined threat to one's existence (Jin & Ryu, 2021; Templer et al., 2006). The second part of the combined index captures fear of Covid-19. This index reflects to what extent respondents felt acutely anxious due to the Covid-19 pandemic.^3^ The Cronbach’s alpha for this combined index is 0.84; higher values of this variable indicate higher affectedness in mental well-being by the pandemic.

Additional independent variables were included as control variables:

- *donated in 2019* (1 = donated in 2019, 0 = did not donate in 2019). The variable is only included in models 1 and 3 (dependent variable: increase in donations), as all individuals with decreased donations (models 2 and 4) by definition donated in 2019.
- *age* (ref. category age 30 or younger)
- *gender* (1 = female, 0 = male)
- *education* (1 = finished upper secondary education or higher, 0 = did not finish upper secondary education)
- *rural/urban residence* (1 = rural, 0 = urban)
- *country of survey* (1 = Austria, 0 = Germany)
- *adjusted household income* per capita (monthly net disposable household income, in Euro)^4^
- *religiosity* (4-point Likert scale, ranging from 1—“not religious” to 4—“very religious”, treated as if metric)
- *altruism*^5^ (index, from 1—“not altruistic”, to 4—“very altrustic”, treated as if metric)
- *generalized social trust*^6^ (7-point Likert scale, ranging from 1—“low generalized social trust”, to 7—“high generalized social trust”, treated as if metric).

All metric variables were standardized (to *μ* = 0 and *σ* = 1). Those with missing data on one or more responses were excluded from the analyses. The share of missing data in two variables (household income and religiosity) was relatively high. To test the robustness of the reported results, we performed a bootstrapping procedure (see below). Through repeatedly drawing random samples from the data, it can be ensured that the results are not driven by the patterns of missing data. Table 4 in "Appendix" presents a descriptive overview of all independent variables.^7^

## Results

Table 1 displays the results of the first set of logistic regression analyses. The dependent variable is “increase in donations” in Models 1 and 3 and “decrease in donations” in Models 2 and 4. The reference category in all models is “no change in donations.” The results reveal that individuals that were negatively affected by the pandemic mentally had a significantly higher likelihood of displaying changes in their charitable giving behavior, which is in-line with our expectations. Interestingly, increased mental affliction is associated with both higher and lower levels of donations. Furthermore, being negatively affected by the crisis in financial terms makes people more likely to decrease their donations, while no significant relation was found with increases in donations (see models 1 and 3). Being negatively affected health-wise by the pandemic revealed no significant relation with the two dependent variables.

**Table 1:** Results of the logistic regression (whole sample)

	Dependent variable:			
	+ donate	− donate	+ donate	− donate
	(Mod 1)	(Mod 2)	(Mod 3)	(Mod 4)
Personal affliction—mentally	0.262***	0.198**	0.280***	0.212**
	(0.082)	(0.086)	(0.083)	(0.087)
Personal affliction—financially	0.057	0.278***		
	(0.083)	(0.087)		
Personal affliction—health-wise			− 0.061	0.093
			(0.081)	(0.086)
Donated in 2019	− 0.503**		− 0.502**	
	(0.220)		(0.220)	
Female	0.192	− 0.042	0.199	− 0.028
	(0.166)	(0.174)	(0.165)	(0.174)
Age: 31–45	0.275	− 0.067	0.279	− 0.040
	(0.253)	(0.245)	(0.253)	(0.244)
Age: 46–60	− 0.106	− 0.362	− 0.087	− 0.307
	(0.263)	(0.254)	(0.262)	(0.253)
Age: 61 +	0.219	− 0.351	0.235	− 0.342
	(0.249)	(0.250)	(0.249)	(0.249)
A levels	0.218	0.268	0.217	0.239
	(0.180)	(0.186)	(0.181)	(0.185)
Rural Area	0.067	0.022	0.069	0.035
	(0.164)	(0.173)	(0.164)	(0.172)
Austria	0.222	0.284	0.231	0.307*
	(0.165)	(0.175)	(0.165)	(0.174)
Household income p.c	0.260***	0.039	0.240**	− 0.002
	(0.095)	(0.095)	(0.094)	(0.093)
Religiosity	0.239***	0.077	0.238***	0.077
	(0.081)	(0.084)	(0.081)	(0.084)
Altruism	0.140	0.378***	0.140	0.384***
	(0.088)	(0.094)	(0.088)	(0.093)
Gen. Social trust	0.101	0.187**	0.087	0.162*
	(0.086)	(0.091)	(0.085)	(0.090)
Constant	− 2.242***	− 2.101***	− 2.264***	− 2.114***
	(0.265)	(0.261)	(0.265)	(0.261)
Observations	1353	1345	1353	1345
Log Likelihood	− 517.093	− 476.352	− 517.049	− 480.904
Akaike Inf. Crit	1064.185	980.705	1064.098	989.808

*p < 0.1; **p < 0.05; ***p < 0.01

Regarding the control variables, we find that individuals who donated money in 2019 were less likely to increase donations during the pandemic. A higher household income and being religious are associated with a higher likelihood of increasing donations. Turning to factors associated with decreased donations, we find that individuals with higher levels of altruism were more likely to decrease donations. Similarly, individuals with a higher generalized social trust were more likely to decrease their donations, although the coefficients are statistically significant on a 5%- (model 2) or 10%- (model 4) level only. Austrian respondents were also more likely to decrease donations compared to their German counterparts. Again, these coefficients were statistically significant on a 10%- (model 2) or 5%- (model 4) level only. The remaining control variables did not reveal any statistically significant relationship with the dependent variables.

In order to analyze differences between those who increased donations and those who decreased them, we estimated further models using a reduced sample that included only respondents who changed their charitable giving behavior during the Covid-19 pandemic. The logistic regression models displayed in Table 2 contrast respondents who donated more money to those who donated less, i.e., the dependent variable is coded 0 for a decrease in donations and 1 for an increase in donations, with Model 5 including mental and financial affliction, and Model 6 including physical health. While we find no impact of mental well-being on the dependent variable, both financial and health-wise affliction help explaining whether a person was more likely to donate more or less. More precisely, respondents who reported that they were afflicted financially or in their physical health were less likely to increase donations. However, this finding is statistically significant on a 10%-level only, which can probably (at least partly) be explained by the rather small sample size in these regressions.

**Table 2:** Results of the logistic regression (reduced sample)

	Dependent variable:	
	+ donate	
	(Mod 5)	(Mod 6)
Personal affliction—mentally	0.101	0.117
	(0.112)	(0.112)
Personal affliction—financially	− 0.194*	
	(0.110)	
Personal affliction—health-wise		− 0.078
		(0.060)
Female	0.227	0.185
	(0.220)	(0.220)
Age: 31–45	0.368	0.287
	(0.328)	(0.328)
Age: 46–60	0.266	0.221
	(0.342)	(0.340)
Age: 61 +	0.518	0.491
	(0.329)	(0.328)
A levels	− 0.207	− 0.221
	(0.232)	(0.232)
Rural Area	0.077	0.056
	(0.222)	(0.221)
Austria	− 0.271	− 0.261
	(0.225)	(0.225)
Household income	0.186	0.213
	(0.137)	(0.136)
Religiosity	0.188*	0.187*
	(0.108)	(0.108)
Altruism	− 0.233*	− 0.245**
	(0.123)	(0.123)
Gen. Social trust	− 0.110	− 0.109
	(0.122)	(0.122)
Constant	− 0.172	0.284
	(0.339)	(0.485)
Observations	366	366
Log Likelihood	− 244.079	− 244.814
Akaike Inf. Crit	516.159	517.628

*p < 0.1; **p < 0.05; ***p < 0.01

Turning to the control variables, we find that more religious respondents were more likely to be in the group that gave more money to charitable causes. More altruistic individuals were less likely to be in this group. Again, most results are only weakly statistically significant.

In addition to the analyses presented in the results section, several robustness checks were performed. First, models 1 to 4 were estimated separately for the Austrian and German samples to investigate whether the relationships found between the variables are consistent between the two countries. Overall, the results (see online supplementary material) reveal no major differences between the two countries, which suggests that the effects apply across countries. Some minor differences, however, are visible. Respondents from Germany who stated that they were affected financially were more likely to change their charitable giving behavior, both decreasing and increasing donations (with the effect on decreasing donations being larger), while Austrians who were affected financially were only more likely to decrease donations. Turning to physical health, we find that Austrians who stated that they were affected were less likely to increase donations, while this coefficient was not significant in the German sample.

As a second robustness check, we re-estimated models 1 to 4 with 1000 randomly drawn balanced samples (“bootstrapping”). We over-sampled the cases where individuals changed their charitable giving behavior to produce 1000 balanced random samples in which each 1000 individuals increased and decreased their charitable giving, and compared those to 1000 individuals that did not change their charitable giving. As mentioned above, balancing the samples usually increases the fit of the theoretical model to the data (Salas-Eljatib et al., 2018). All results as reported above also hold for the balanced samples: e.g., in more than 950 out of 1000 samples, personal mental affliction is associated with changes in donation behavior (*α* = 0.1).

## Discussion

How does a severe global crisis like the Covid-19 pandemic affect individuals’ charitable giving behavior? Provocatively, we asked whether the Covid-19 pandemic brought out the better or worse in people. First of all, our findings reveal that during the Covid-19 crisis, most individuals did not change their charitable giving. More than three-quarters of the respondents did not alter their giving, and comparatively few persons increased or decreased their giving. Although this small proportion could question the relevance of the study itself, this finding is crucial as it shows that even a shock as severe as the Covid-19 pandemic does not alter giving behavior in a substantial way. This is in-line with prior findings that participation rates in a country do not fluctuate substantially over time. Moreover, our findings are consistent with changes in giving behavior found in other countries during the pandemic, including countries with other welfare traditions such as Australia and the US, as well as Sweden (Wiepking et al., 2023).

Still, there are respondents who alter their behavior, and either increase or decrease their charitable giving. We find that these changes can be partly explained by the factors that also predict giving in normal times: Individuals who are more religious, who live in higher income households, or who are over 60 years old were more likely to increase their giving during the pandemic (Neumayr & Schober, 2012; Wilke, 2020). Moreover, our analyses show that being affected on a personal level plays an important role in predicting behavioral changes. The observed patterns seem similar to previous studies which showed that the closer someone is to a societal problem, the more likely the person donates for the given issue (Lim & DeSteno, 2016; Piff et al., 2010).

In which ways has personal affectedness by the crisis changed giving behavior? Respondents who stated to be concerned in their mental or financial well-being were more likely to change their donations compared to those less concerned on their mental or financial well-being, as hypothesized. The direction of change, i.e., whether people increased or decreased their donations, depends on the type of concern. While being mentally affected leads to increases as well as decreases in donations, financial and health-wise afflictions are associated primarily with a decrease in donations.

The impact of the pandemic’s mental well-being effects on charitable giving behavior in both directions can be explained with the nuances of TMT (Pyszczynski et al., 2021). On the one hand, some individuals reduced their donations, which according to TMT is caused by efforts to repress or process the acute danger (proximal defenses). On the other hand, there was an increase in donations, which TMT attributes to an increased need to give meaning to one's own life and an increased orientation toward the social norms of the surrounding culture (distal defenses). About half of those that were severely mentally affected during the first months of the pandemic seem to have been able to cope with the existential threat by mid-2021 (Hettich et al., 2022; Lueger-Schuster et al., 2022)—the distal defenses prevailed for them.^8^

If individuals were financially or physically affected, they seem to have been mostly overwhelmed by the situation and not able to react in prosocial manner: They stayed in a state were self-preservation was the priority (proximal defenses), thus not being able to also attend to the needs of others. These findings, together with the higher likelihood of high-income households to increase their donations, indicate that financial conditions play a vital role in individuals’ abilities to process existential threats. Financial and health insecurity seem to leave individuals stuck in the proximal defense mode, struggling for survival, not able to productively channel the experiences in the form of distal defense mechanisms (Claes et al., 2021; Kopasker et al., 2018).

Although we report only on changes in charitable giving, we think that the patterns we see there convey an idea of changes in overall pro-sociality, as giving is positively correlated to other forms of prosocial behavior (Bauer et al., 2013). Prior research reports increases in giving for the first months of the pandemic for the US (Fridman et al., 2022), and investigated crises’ consequences for overall solidarity, public support (Koos, 2019; Prainsack, 2020), and on overall social inequality and individual well-being (Grasso et al., 2021). We contribute to this research by introducing TMT to explain the changes theoretically and conceptually.

Our study draws attention to a possible blind-spot of previous studies investigating the consequences of existential threats on prosocial behavior: As preceding studies were conducted in experimental settings, the participants were mostly able to cope with the threat and thus act from distal defense mode. Participants tried to validate their worldview and acted pro-socially. A real-world threat like Covid-19, however, makes it much harder to suppress the threat. Many people were not able to move from proximal defenses to distal defenses. They kept occupied with caring for their own survival during the first year of the pandemic. Hence, the positive effect of existential threats on prosocial behavior seems to be more limited in real-world scenarios compared to experimental setting, as people cannot just step out of the threating situation.

However, as a limitation, we would like to address the limited sample size that prevented a more detailed analysis differentiating between changes in incident and amount. Additionally, a bigger sample size could be used to analyze potential changes to choices among causes of donors. We would have liked to receive much more fine-grained data on, e.g., the particular amounts donated to specific causes. As a second limitation, we want to point out that our questionnaire-based data reveal the limits of asking for ex-post accounts of very concrete actions. It is plausible that respondents failed to remember accurately over a timespan of two years. Consequently, individuals’ information on their charitable giving could be under- or overestimated.

It is important to note that our model considers context factors only by including a country-fixed effect. Clearly, we thus cannot identify relevant contextual factors that might help explain a behavioral change in a differentiated way. Importantly, such contextual factors may have changed in the course of the pandemic as well. For instance, savings rates have risen during the crisis, which could have had an impact on donations. Multi-level analysis would be required to adequately account for more contextual factors, and a comparative analysis of more than just two countries would help single out important contextual factors.

## Conclusion

What do our results suggest about prosocial behavior in our contemporary societies, haunted by crisis after crisis? For once, we see that few people changed their behavior in the face of such a severe and visible crisis as Covid-19. We deem this result as comparatively good news for nonprofit organizations that rely on donations as their main income source. The pandemic did not lead to drastic changes in the donation patterns, and the group that decreased donations is about the same size as the one that increased donations. Being personally affected makes behavioral changes much more likely. This is in-line with previous research on giving in the wake of disasters. Understandably, those who suffered financial losses due to the Covid-19 crisis or were affected health-wise more often were in the group that decreased donations.

### Electronic supplementary material

Below is the link to the electronic supplementary material.

Supplementary file 1

## Appendix

See Tables 3 and 4.

**Table 3:** Dependent variable: changes in incidence and amount of donations

	Donated 2019	Donated 2020/21	*N*	Valid %
Increase in donations (Likelihood)	0	1	192	9.7
Increase in donations (amount donated)	1	1	46	2.3
Increase in donations total			238	12.0
Decrease in donations (Likelihood)	1	0	193	9.8
Decrease in donations (amount donated)	1	1	19	1.0
Decrease in donations total			212	10.7
No change in donations	0	0	1223	61.9
No change in donations (amount donated)	1	1	303	15.3
No change in donations total			1526	77.2
Valid total			1976	100
Missing			24

**Table 4:** Descriptives of variables (unstandardized); *n* = 2000

	Frequency	Valid %	Mean	Median	SD	Min	Max
Personal affliction—mentally	2000	100	2.46	2.40	0.77	1.00	5.00
Personal affliction—financially	2000	100	4.50	5.00	2.01	0.00	10.00
Personal affliction—health-wise	2000	100	4.75	5.00	1.78	0.00	10.00
Donated in 2019	1981	100					
Yes	566	29.2					
No	1415	70.8					
Missing	19	–					
Age	2000	100	48.5	50.0	16.4		
18–30	379	19.0					
31–45	486	24.3					
46–60	567	28.4					
61+	568	28.4					
Gender	1999	100					
Female	1028	51.4					
Male	971	48.6					
Missing	1	–					
Educational background	2000	100					
Below A level	1343	67.2					
A level	657	32.9					
Location	2000	100					
Urban	1054	52.7					
Rural	946	47.3					
Adjusted household income per capita	1750	87.5	1.857€	1767€	949€	150€	8485€
missing	250	–					
Religiosity	1765	88.3	1.92	2.00	1.07	1	4
missing	235	–					
Altruism	1983	99.1	2.80	2.67	0.72	1	4
missing	17	–					
Social trust	2000	100	4.00	4.00	1.51	1	7

## Footnotes

1 Death anxiety items: “I am worried about my health”; “I am scared at thoughts of death”; “I'm afraid I might die soon”; “When I read or hear about an illness, I feel its symptoms”; “If I notice an unfamiliar physical sensation, I get worried quickly”.

2 Fear of Covid-19 items: “During the pandemic, I have felt moments of fear, anxiety or panic due to Covid”; “During the pandemic, I have felt tense, restless, or had troubles relaxing because of the coronavirus”; “During the pandemic, I have avoided certain situations”; “During the pandemic, I have distracted myself so as not to think about Corona”; and “During the pandemic, I have needed help to get through certain situations (e.g. through using medication, other people)”.

3 These items were taken from a large German social science panel project, https://www.soscipanel.de/.

4 Disposable income per member of the household is not directly proportional to the number of household members due to economies of scale. Thus, we used an equivalence scale (square root scale) to compute adjusted values, following the most recent method as used by the OECD, see https://www.oecd.org/els/soc/OECD-Note-EquivalenceScales.pdf, last accessed 09.03.2022.

5 Altruism was measured on a four-point Likert scale ranging from 1 “completely agree” to 4 “completely disagree” based on three items originally from the Survey of Interpersonal Values (SIV). The items read (1) “I try to work towards the wellbeing of society”, (2) “It is important to me that I help others”, (3) “I think it is important to help the poor and the needy”. The scale was recoded so that the value 4 indicates a very altruistic individuum.

6 Following Lundmark et al. (2016), participants' responses to the question “In your opinion, to what extent is it generally possible to trust people?” on a seven-point scale ranging from 1 “People cannot generally be trusted” to 7 “Most people can be trusted”.

7 All data available upon request.

8 It need to be mentioned that incidence of mental affliction like anxiety and depression went up again in late 2021 and early 2022, when the number of infections were rising again and restrictions more severe (Hettich et al., 2022; Lueger-Schuster et al., 2022).
