# Supplementary material for: Charitable Giving in Times of Covid-19: Do Crises Forward the Better or the Worse in Individuals?
Source: Voluntas. 2023 Feb 28:1–13. Online ahead of print. doi: 10.1007/s11266-023-00558-y (PMC9974051; doi:10.1007/s11266-023-00558-y)
Supplement: Supplementary file 1 [file 11266_2023_558_MOESM1_ESM.docx]

Online supplementary material

Table S1. Results of the logistic regression - Austria only

=========================================================================

Dependent variable:

---------------------------------------

+ donate - donate + donate - donate

(Mod 1) (Mod 2) (Mod 3) (Mod 4) -------------------------------------------------------------------------

Personal affliction – mentally 0.408*** 0.213* 0.472*** 0.224*

(0.118) (0.124) (0.123) (0.128)

Personal affliction - financially -0.144 0.227*

(0.119) (0.124)

Personal affliction - health-wise -0.293** 0.084

(0.118) (0.124)

Donated in 2019 -0.666** -0.672**

(0.283) (0.285)

Female 0.071 -0.195 0.023 -0.171

(0.228) (0.236) (0.230) (0.235)

Age: 31-45 1.071*** 0.171 1.040*** 0.202

(0.392) (0.337) (0.392) (0.335)

Age: 46-60 0.499 -0.371 0.484 -0.337

(0.409) (0.362) (0.408) (0.361)

Age: 61+ 1.291*** 0.082 1.331*** 0.082

(0.389) (0.342) (0.391) (0.342)

A levels 0.123 0.039 0.116 0.021

(0.247) (0.249) (0.247) (0.249)

Rural Area 0.113 0.078 0.116 0.100

(0.224) (0.230) (0.225) (0.229)

Household income p.c. 0.186 -0.093 0.172 -0.140

(0.141) (0.141) (0.140) (0.137)

Religiosity 0.184* 0.036 0.159 0.038

(0.111) (0.113) (0.112) (0.113)

Altruism 0.150 0.305** 0.136 0.311**

(0.121) (0.126) (0.122) (0.126)

Gen. Social trust 0.061 0.324*** 0.056 0.314**

(0.118) (0.125) (0.118) (0.125)

Constant -2.627*** -1.856*** -2.618*** -1.864***

(0.402) (0.345) (0.400) (0.346)

-------------------------------------------------------------------------

Observations 694 672 694 672

Log Likelihood -273.111 -259.564 -270.749 -261.022

Akaike Inf. Crit. 574.223 545.127 569.497 548.044

=========================================================================

Note: *p<0.1; **p<0.05; ***p<0.01

Table S2. Results of the logistic regression - Germany only

=========================================================================

Dependent variable:

---------------------------------------

+ donate - donate + donate - donate

(Mod 1) (Mod 2) (Mod 3) (Mod 4) -------------------------------------------------------------------------

Personal affliction – mentally 0.197 0.315** 0.202* 0.326**

(0.122) (0.130) (0.121) (0.129)

Personal affliction - financially 0.282** 0.333**

(0.125) (0.130)

Personal affliction - health-wise 0.140 0.108

(0.122) (0.125)

Donated in 2019 -0.436 -0.430

(0.364) (0.362)

Female 0.414* 0.057 0.420* 0.067

(0.251) (0.273) (0.251) (0.272)

Age: 31-45 -0.443 -0.374 -0.410 -0.380

(0.358) (0.382) (0.358) (0.382)

Age: 46-60 -0.650* -0.403 -0.576 -0.337

(0.365) (0.377) (0.362) (0.375)

Age: 61+ -0.840** -0.997** -0.818** -0.984**

(0.362) (0.413) (0.362) (0.411)

A levels 0.265 0.513* 0.240 0.454

(0.272) (0.293) (0.273) (0.291)

Rural Area 0.089 0.055 0.096 0.038

(0.250) (0.278) (0.249) (0.276)

Household income p.c. 0.361*** 0.146 0.342** 0.123

(0.135) (0.136) (0.134) (0.134)

Religiosity 0.271** 0.035 0.266** 0.028

(0.123) (0.136) (0.122) (0.135)

Altruism 0.151 0.448*** 0.154 0.455***

(0.134) (0.146) (0.132) (0.145)

Gen. Social trust 0.212 0.047 0.173 0.007

(0.133) (0.143) (0.130) (0.140)

Constant -1.780*** -2.086*** -1.800*** -2.059***

(0.341) (0.364) (0.342) (0.359)

-------------------------------------------------------------------------

Observations 659 659 659 659

Log Likelihood -230.065 -199.443 -231.969 -202.414

Akaike Inf. Crit. 488.130 424.887 491.938 430.828

=========================================================================

Note: *p<0.1; **p<0.05; ***p<0.01

S3. Questionnaire - Prosocial behavior in times of Corona

The following questionnaire was translated from German to English for the sake of scientific replicability. The questions in the form below were asked in German only during the original survey, conducted in Germany and Austria in September 2021.
Only the items that were eventually used for publication were translated.

Green: Questions not translated into English because they were not used in the models presented in the article
Grey: Notes for programming

Cyan: Open answers

# Questions regarding prosocial behavior

Introduction to survey:

*This survey is about donations and volunteering.*

*This can take many forms: There are people who donate money, or things like clothes. For example, for international projects, for cancer research, at collections in church or to people on the street. Other people volunteer in an organization like the Red Cross or the volunteer fire department, bake cakes for events or get involved in the parents' association. Some directly support people in the neighborhood. And many also choose not to get involved in any of the above for a number of good reasons.*

*Your information will support the research, no matter which group you belong to!*

- 1. Try to remember the time before Corona erupted, the still 'normal' year 2019. Please tick which of the following activities you did in 2019.

[show first answer column only]

Now think about the time after Corona erupted in March 2020. Which of these activities have you done since Corona erupted? [show all columns]:

|  | In 2019 | Since the eruption of Corona between March 2020 and February 2021 | Currently in September 2021 | In none of the periods mentioned |
| --- | --- | --- | --- | --- |
| 1. Volunteered in an organization [-> if yes, question 1.2] | □ | □ | □ | □ |
| 1. Donated money to an organization [-> if yes, question 1.3] | □ | □ | □ | □ |
| 1. Donated blood or plasma | □ | □ | □ | □ |
| 1. Food-donations or donations in kind to an organization | □ | □ | □ | □ |
| 1. Gave money to strangers in an emergency situation | □ | □ | □ | □ |
| 1. Helped friends, acquaintances or neighbors in an emergency situation | □ | □ | □ | □ |
| 1. Helped strangers in an emergency situation | □ | □ | □ | □ |
| 1. Supporting someone with e.g. going shopping, walking the dog | □ | □ | □ | □ |

[1.2. Follow-up questions regarding volunteering]

1.3 [Follow-up question to 1.1b donations (in money). Show only those columns that were checked at 1.1.b]:
You indicated that you donated money to an organization. In which of the following areas are the organizations you donated to engaged in?

Please select all that apply.

| Donated money… | In 2019 | Since the eruption of Corona between March 2020 and February 2021 | Currently in September 2021 |
| --- | --- | --- | --- |
| Health | □ | □ | □ |
| Religion | □ | □ | □ |
| Social services (Old age, poverty, people with disabilites, youth, volunteer fire department…) | □ | □ | □ |
| International aid | □ | □ | □ |
| Human rights and advocacy | □ | □ | □ |
| Culture and arts | □ | □ | □ |
| Education and research | □ | □ | □ |
| Environment protection and animal help | □ | □ | □ |
| Neighborhood groups, housing and infrastructure projects | □ | □ | □ |
| Sports and social clubs | □ | □ | □ |
| Other [please name]: | □ | □ | □ |

1.3.1 [Show only the areas ticked at 1.3 in 2019 and currently in September 2021]:
How much do you estimate you have donated or expect to donate to each area?

|  | In the whole year of 2019 | In the whole year of 2021 |
| --- | --- | --- |
| Health |  |  |
| Religion |  |  |
| Social services (Old age, poverty, people with disabilites, youth, volunteer fire department…) |  |  |
| International aid |  |  |
| Human rights and advocacy |  |  |
| Culture and arts |  |  |
| Education and research |  |  |
| Environment protection and animal help |  |  |
| Neighborhood groups, housing and infrastructure projects |  |  |
| Sports and social clubs |  |  |
| Other [please name]: |  |  |

[If no amount is mentioned, the following categories are shown:]

1. Less than 10 Euro
2. 11 to 25 Euro
3. 26 to 50 Euro
4. 51 to 100 Euro
5. 101 to 200 Euro
6. 201 to 500 Euro
7. More than 500 Euro
8. Don’t know/ prefer not to say

1.3.2 [Show only those areas that were checked at 1.3 in 2019 AND 2020]:

You indicated that you donated money to organizations in the following areas both before and after the Corona outbreak.

Please estimate: Did you donate less, the same, or more in each area after the Corona outbreak than you did in 2019?

|  | Donated less | Donated an equal amount | Donated more |
| --- | --- | --- | --- |
| Health | □ | □ | □ |
| Religion | □ | □ | □ |
| Social services (Old age, poverty, people with disabilites, youth, volunteer fire department…) | □ | □ | □ |
| International aid | □ | □ | □ |
| Human rights and advocacy | □ | □ | □ |
| Culture and arts | □ | □ | □ |
| Education and research | □ | □ | □ |
| Environment protection and animal help | □ | □ | □ |
| Neighborhood groups, housing and infrastructure projects | □ | □ | □ |
| Sports and social clubs | □ | □ | □ |
| Other [please name]: | □ | □ | □ |

[Questions regarding receiving help during Covid]

# Questions about values and attitudes

Next are some questions about your personal attitudes and opinions. There are no right or wrong answers - don't think about it too long, just tick off what goes through your mind first.

2.1 For each of the following statements, please indicate the extent to which it applies to you personally. [items randomized].

|  | Applies fully |  |  | Does not apply at all | Don’t know |
| --- | --- | --- | --- | --- | --- |
| I try to work for the good of society. | □ | □ | □ | □ | □ |
| Helping others is important to me. | □ | □ | □ | □ | □ |
| I think it is important to help the poor and needy. | □ | □ | □ | □ | □ |
| I am in control of my own life. | □ | □ | □ | □ | □ |
| Whether in my private life or at work: My life is largely determined by others. | □ | □ | □ | □ | □ |
| My plans are often thwarted by fate. | □ | □ | □ | □ | □ |
| If I make an effort, I will also succeed. | □ | □ | □ | □ | □ |
| In a dispute I always remain factual and objective. | □ | □ | □ | □ | □ |
| Even when I am stressed myself, I always treat others in a friendly and courteous manner. | □ | □ | □ | □ | □ |
| When I talk to someone, I always listen to them carefully. | □ | □ | □ | □ | □ |
| Religion is important in my life. | □ | □ | □ | □ | □ |
| [inequality aversion I] |  |  |  |  |  |
| [inequality aversion II] |  |  |  |  |  |
| [warm-glow] |  |  |  |  |  |

2.2. How much do you think you can trust other people?

|  | 1 | 2 | 3 | 4 | 5 | 6 | 7 |  |
| --- | --- | --- | --- | --- | --- | --- | --- | --- |
| In general, you cannot trust other people | □ | □ | □ | □ | □ | □ | □ | In general, most people can be trusted. |
| In general, people cannot be trusted where I live. | □ | □ | □ | □ | □ | □ | □ | In general, most people where I live can be trusted. |

[2.3 & 2.4. Life-satisfaction]

[2.5 How threatening or liberating was your personal experience of the Corona period?]

- 1. How would you say the Corona crisis has affected you personally?

|  | 0 very negatively | 1 | 2 | 3 | 4 | 5 not at all | 6 | 7 | 8 | 9 | 10 very positively |
| --- | --- | --- | --- | --- | --- | --- | --- | --- | --- | --- | --- |
| Overall | o | o | o | o | o | o | o | o | o | o | o |
| Financially | o | o | o | o | o | o | o | o | o | o | o |
| Health-wise | o | o | o | o | o | o | o | o | o | o | o |

- 1. [Moral values]
  2. During Covid, I …

|  | never |  |  |  | constantly |
| --- | --- | --- | --- | --- | --- |
|  |  |  |  |  |  |
| Had moments of terrible fear, anxiety or panic felt due to Corona. | o | o | o | o | o |
| Felt tense or restless due to Corona, or had difficulty relaxing. | o | o | o | o | o |
| Avoided or left certain situations, e.g., crowds. | o | o | o | o | o |
| Distracted myself to avoid thinking about Corona. | o | o | o | o | o |
| Needed help to get through certain situations (e.g., medications, other people). | o | o | o | o | o |

- 1. For each of the following statements, please indicate the extent to which it applies to you personally.

|  | never |  |  |  | most of the time |
| --- | --- | --- | --- | --- | --- |
|  |  |  |  |  |  |
| I am worried about my health. | o | o | o | o | o |
| I am afraid at thoughts of death. | o | o | o | o | o |
| I'm afraid I might die soon. | o | o | o | o | o |
| When I read or hear about a disease, I feel its symptoms. | o | o | o | o | o |
| When I notice an unusual physical sensation, I quickly become concerned. | o | o | o | o | o |

- 1. [Attitudes on income and wealth distribution]

# Sociodemographics

3.1 [Age of respondent]

3.2 [Size and composition of household]

3.3 [Respondent in a relationship/married?]

3.4 [Respondent’s highest educational level attained]

3.5 & 3.6. [Respondent’s employment status; changes during Covid?]

3.7 [At risk of poverty?]

3.8 [Net household income]

3.9 [Political orientation: left-right]

3.10 [Migration background]

3.11. [Zip-code]

3.12 [Gender]
